# Cutaneous α-Synuclein Pathology as a Differential Marker: A Histological and Statistical Comparison across Neurodegenerative Disease Groups

**DOI:** 10.1007/s12031-026-02486-0

**Published:** 2026-02-21

**Authors:** Dorota Šebelová, Kateřina Menšíková, Michaela Kaiserová, Lenka Satke, Zuzana Grambalová, Kateřina Čížková, Zdeněk Tauber, Kateřina Klíčová, Dominik Hraboš, Sarah E. V. Cook, Jana Zapletalová, Petr Kaňovský

**Affiliations:** 1 Department of Neurology, Faculty of Medicine and Dentistry, Palacky University, Olomouc, 77900 Czech Republic; 2 Department of Neurology, Olomouc University Hospital, Olomouc, 77900 Czech Republic; 3 Department of Histology and Embryology, Faculty of Medicine and Dentistry, Palacky University, Olomouc, 77900 Czech Republic; 4 Department of Molecular Pathology, Faculty of Medicine and Dentistry, Palacky University, Olomouc, 77900 Czech Republic; 5 Olomouc Brain Bank, Department of Neurology and Department of Clinical and Molecular Pathology, University Hospital Olomouc and Faculty of Medicine and Dentistry, Palacky University, Olomouc, Czech Republic; 6 Department of Medical Biophysics, Olomouc University Hospital, Olomouc, Czech Republic

**Keywords:** Skin biopsy, Biomarkers, Neurodegenerative diseases, Α-synucleinopathies, Tauopathies, Parkinson’s disease

## Abstract

There is an urgent need for early and accurate biomarkers of neurodegenerative disorders. Due to the high innervation and accessibility of the skin, a skin biopsy is a minimally invasive method of detecting phosphorylated α-synuclein (p-α-syn) and assessing intraepidermal nerve fiber density (IENFD). We analyzed biopsies taken from the back and the leg of patients with parkinsonian syndromes (Park.sy.), α-synucleinopathies, multiple system atrophies (MSA), tauopathies, and other neurological disorders, as well as from healthy controls. Double immunofluorescence was performed for p-α-syn (Ser129) and protein gene product 9.5 (PGP 9.5), alongside quantitative IENFD assessment. p-α-syn was significantly more prevalent in the patient groups than in the control group. The highest prevalence was observed in patients with parkinsonian syndromes, α-synucleinopathies and MSA. Tauopathies showed preferential paravertebral positivity. Reduction or absence of IENFD was most pronounced in tauopathies (75%), while IENFD was most commonly preserved in MSA (83.3%), indicating that disease-specific patterns of peripheral nerve involvement are exhibited. p-α-syn positivity was found to correlate with shorter disease duration, suggesting its potential as an early biomarker. Combined with olfactory testing, cutaneous markers improved diagnostic discrimination. Our findings support the use of skin biopsies as a promising clinical tool in diagnosing biomarker-based neurodegenerative diseases.

## Introduction

Skin biopsy appears to be a highly sensitive (> 80%) and specific (close to 100%) clinical tool for diagnosing α-synucleinopathies. This minimally invasive outpatient test presents a low risk of adverse effects and is painless. The skin is a promising organ for searching biomarkers because it shares an embryological origin (ectoderm) with nerve tissue. It is also highly innervated and easily accessible. Moreover, it can be used for single or repeated sampling, and environmental and aging changes are reflected by its fibroblasts (Gopar-Guevaz et al. 2021).

This work aims to clarify the diagnostic value of skin biopsies in α-synucleinopathies and determine if skin-derived pathological features can reliably differentiate these disorders. Specifically, this review seeks to: (1) evaluate the usefulness of skin biopsies for diagnosing Parkinson’s disease and related α-synucleinopathies, (2) summarize the methodological factors influencing the detection of phosphorylated α-synuclein in skin tissue, and (3) assess how additional cutaneous markers, such as intraepidermal nerve fiber density, contribute to diagnostic accuracy and disease characterization.

### Skin Sample Collection and Histological Examination

The choice of biopsy site can directly affect test sensitivity. In most studies, the leg and paravertebral areas were the sites of choice (Waqar et al. 2023). Regardless of the body sampling site, an average skin biopsy for α-synuclein (α-syn) aggregate detection is 3–5 mm. This size ensures the biopsy includes the epidermis and dermis, covers areas with the greatest innervation, and provides sufficient tissue (25–100 mg) for subsequent analysis (Carrazana et al. 2025). Autonomous substructures are not always detectable in skin tissue samples, which can be explained by the hypothesis that thicker Sect. (50 μm) increase the detection rate of phosphorylated α-synuclein (p-α-syn) in skin biopsies compared to thinner (10 μm) sections. Thicker tissue sections tend to preserve the continuity of p-α-syn in nerve fibers.

### Phosphorylated Alpha-synuclein

Pathology of α-syn is closely associated with nerve degeneration. The detection rate of p-α-syn from skin biopsies varies widely across different publications, ranging from 30 to 100% (Tsukita et al. 2019; Li et al. 2024; Gibbons et al. 2023; Melli et al. 2018; Donadio et al. 2017; Doppler et al. 2015; Donadio et al. 2020; Giannoccaro et al. 2022). Significant differences in p-α-syn detection between publications highlight the potential impact of methodological differences on results and varying slice thicknesses ranging from 5 to 50 μm. Nolano et al. (2022) describe that p-α-syn is found predominantly in perivascular nerve fibers, sweat glands, and nerve bundles, and less frequently in pilomotor nerves, subepidermal fibers, and isolated fibers (Zhao et al. 2025). Overall findings suggest that p-α-syn deposits begin to appear as a precursor to nerve degeneration, which is influenced by the severity and rate of disease progression. Further degeneration, alongside the loss of nerve fibers, may be associated with the removal of p-α-syn aggregates by a macrophage-mediated inflammatory response (Kuzkina et al. 2021). The widespread patterns of autonomous p-α-syn deposits are also relevant, ranging from fine granular clear staining to small segments along the fiber and heavy deposits in axonal swellings. These patterns may indicate different stages of the degenerative process, leading to progressive accumulation of aggregates in parallel with visible degenerative aspects. The visualization of p-α-syn deposits in the skin is associated with preserved innervation and slower disease progression (Nolano et al. 2022). α-syn undergoes various post-translational modifications, e.g., phosphorylation, cross-linking, or ubiquitination. These modifications can cause α-syn aggregation and contribute to disease pathogenesis in vivo. Phosphorylation at the serine residue 129 (Ser129) of p-α-syn leads to its aggregation, resulting in increased toxicity and leading to the development of Parkinson´s disease (PD) (Waqar et al. 2023). Studies using immunohistochemical and biochemical techniques have shown that detecting α-syn aggregates in peripheral nerves via skin biopsies is a valuable and effective method for distinguishing between individuals with PD from other forms of parkinsonian syndrome and healthy controls (Carrazana et al. 2025). Recent studies have proposed alternative diagnostic criteria for PD based on biomarkers, known as SynNeurGe (Höllinger et al. 2024), as well as staging criteria for α-synucleinopathies, referred to as NSD-ISS (Simuni et al. 2024; Dam et al. 2024). The SynNeuGe criteria suggest that detecting α-syn in the skin tissue (using immunological methods and seed amplification assays) could serve as a complementary biomarker. However, testing for α-syn in the skin has not yet been included in the NSD-ISS staging criteria. The detection of α-syn in the skin is a minimally invasive and more accessible method, making it a more acceptable choice for patients. Additionally, skin biopsy enables the quantitative detection of α-syn and the identification of specific cell types in which it accumulates, using techniques such as immunofluorescence (IF) and IHC (Donadio et al. 2023). Furthermore, examining skin pathology at multiple sites clarifies the distribution of α-syn in both proximal and distal areas of the limbs, helping to distinguish PD from other α-synucleinopathies (Gibbons et al. 2023). In addition to α-syn deposition, PD is also associated with other peripheral pathological features, including reduced IENFD (Dubbioso et al. 2021; Dabby et al. 2012). Incorporating these various pathological changes into skin pathology may improve PD diagnosis specificity, further supporting the development of biomarker-based diagnostic criteria (Zhao et al. 2025).

Research suggests that the distribution of cutaneous p-α-syn may help differentiate between idiopathic Parkinson´s disease (IPD), multiple system atrophy (MSA), dementia with Lewy bodies (DLB), and pure autonomic failure (PAF) (Waqar et al. 2023; Ehler 2012; Gummerson et al. 2025; Donadio et al. 2016, 2020, 2023; Doppler et al. 2015; Giannoccano et al., 2022).

### Intraepidermal Nerve Fiber Density

Intraepithelial nerve fiber density (IENFD) is the gold standard indicator for quantifying skin innervation loss in the diagnosis of small fiber neuropathy (Jeziorska et al. 2019). Examining IENFD from a skin biopsy is currently one of the key methods in the diagnostic algorithm for neuropathic pain and sensory neuropathies, especially small fiber neuropathy, i.e. a clinical entity characterized by isolated involvement of α-δ and C-type small nerve fibers (Buršová et al. 2012).

Skin biopsy is a minimally invasive method allowing evaluation of morphological changes, particularly the reduction in the number of thin fibers in the epidermis. This examination is unique because it can objectively diagnose damage to thin nerve fibers that cannot be detected by standard electromyography or peripheral nerve conduction studies. From a morphological perspective, the early stages of peripheral neurodegenerative damage are characterized by frequent fiber fragmentation, varicosity formation, and/or changes in branching. These changes also precede the actual reduction of IENFD (Buršová et al. 2012).

IENFD correlates with disease duration, disease severity, and cumulative levodopa dose, but not with autonomic symptoms (Doppler et al. 2014; Kass-Iliyya et al. 2015). The correlation between reduced distal IENFD and disease duration is consistent with axonal transport and retrograde axonal transport impairment, as it suggests slowly progressive nerve fiber degeneration with disease progression (Doppler et al. 2014).

**Fig. 1:**
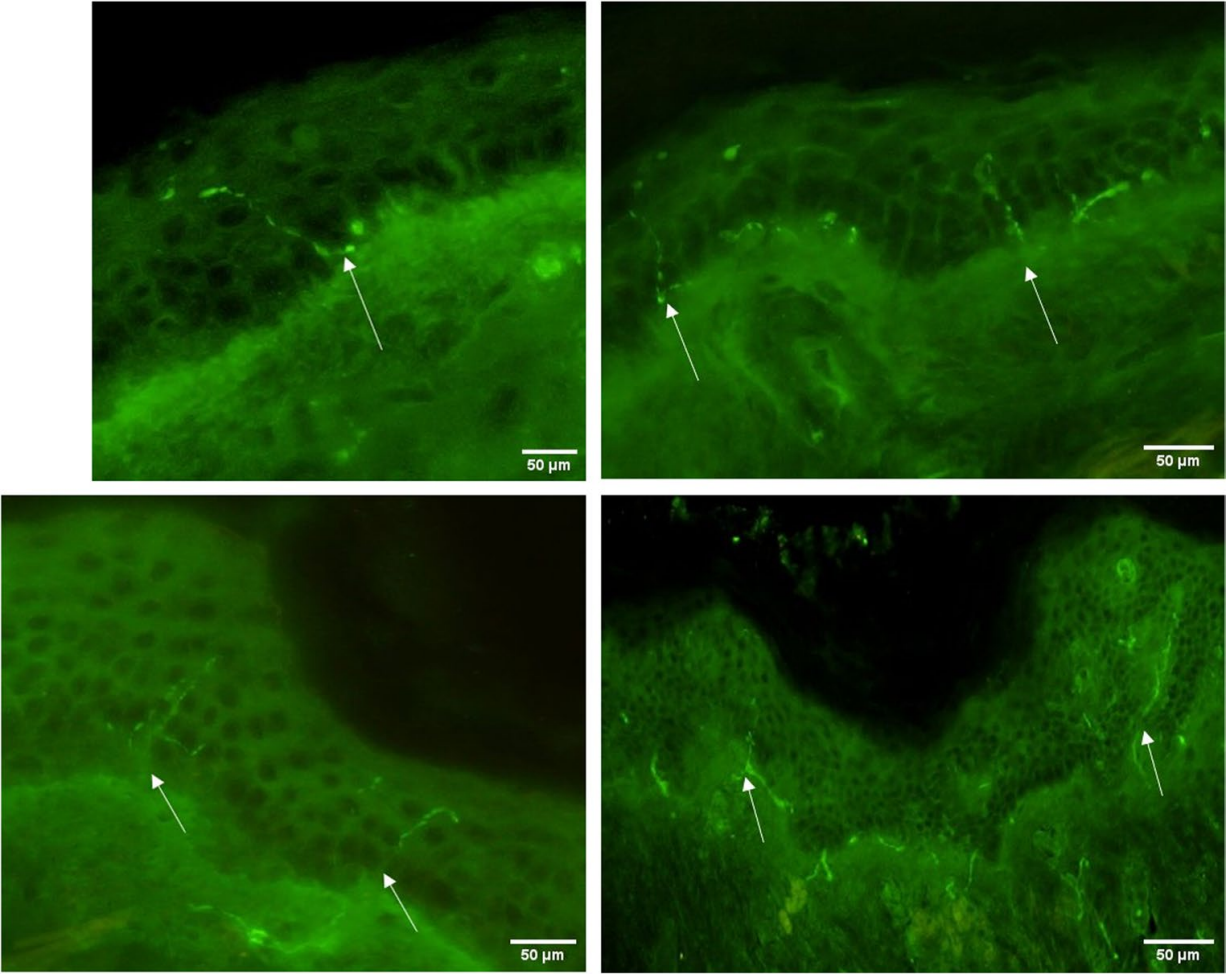
Epidermal innervation visualized by PGP 9.5 immunofluorescence. Microscopic photos of thin fibers (IENFD) (marked with white arrow) in the epidermis. The scale bar is 50 µm and the magnification is 200x.

Skin biopsy with IENFD measurement has become a valuable tool for evaluating patients with neuropathy and neurodegenerative diseases. Two different techniques are employed:

(1) bright-field immunohistochemistry and (2) indirect immunofluorescence, which can be performed with or without confocal microscopy (Lauria et al. 2010; Bakkers et al. 2009). IENFD detection is ensured by the pan-neuronal marker protein gene product (PGP 9.5) (Lauria et al. 2010) (Fig. 1).

Standardization uses normative reference values based on IENFD density in healthy controls from distal leg analyses (Lauria et al. 2010). These values are determined by age and gender (Lauria et al. 2010; Nolano et al. 2015; Provitera et al. 2016; Collongues et al. 2018; Gasparotti et al. 2017; Piscosquito et al. 2021; Kelley and Hackshaw 2021). According to published literature, the normative values for healthy individuals in the distal leg (with a cutting thickness of 50 μm) range from 9.8 ± 3.6/mm to 13.8 ± 6.7/mm (mean ± standard deviation) (Lauria et al. 2005, 2010). The density of intraepidermal nerve fibers decreases in the leg from the proximal thigh to the ankle (Lauria et al. 2010). However, most studies also demonstrate gender- and ethnicity-related differences in IENFD density (Lauria et al. 2010; Bakkers et al. 2009; Collongues et al. 2018; Roglio et al. 2008). Therefore, the authors recommend not taking the patient’s gender into account when using summary standards, as gender-related differences in IENFD density may be related to hormonal status (Buršová et al. 2012). The reasons for the age-related decline in IENFD density are likely related to the physiological processes of aging (Lauria et al. 2010).

## Patients and Methods

The study protocol was approved by the Institutional Ethics Committee of the Faculty of Medicine and Dentistry, Palacky University Olomouc and Olomouc University Hospital. Ethics approval for this study was granted in accordance with the Olomouc University Hospital standard SM-L031, and the ethics committee reference number is 88/21. All patients and healthy controls were informed about the purpose and the design of the study and they all signed informed consent forms. Patient enrollment, data collection, sampling, and laboratory examinations took place between 2022 and 2025. No blinding was performed.

Disease classification was based exclusively on clinically established diagnoses, as pathological confirmation was not feasible due to the use of skin biopsies obtained from living subjects. All diagnoses were determined by experienced neurologists according to currently accepted clinical diagnostic criteria for the respective disorders. The terms “confirmed α-synucleinopathy” and “confirmed tauopathy” therefore refer to clinically confirmed diagnoses and do not imply neuropathological verification.

The patient samples (120) were divided into groups according to diagnoses established using current validated and updated clinical criteria (Alexander et al. 2014; Armstrong et al. 2013; Bhidayasiri et al. 2019; Boxer et al. 2017; Gilman et al. 2008; Höglinger et al. 2017; McKeith et al. 2020; Postuma et al. 2015). Patients were included in the study if they were able to undergo all examinations specified in the study protocol, complied with follow-up visits, and had no serious comorbidities that could interfere with the planned assessments, such as cancer, hematological disorders, depression, or psychosis. The patients diagnosed with proteinopathy were divided into groups based on their clinical diagnoses and clinical-pathological taxonomy:

1. parkinsonian syndromes (Park.sy.) (patients without a confirmed clinical diagnosis; the disease may develop either on the side of α-synucleinopathies or tauopathies) (the cohort contained 33 patients);
2. α-synucleinopathies group (idiopathic Parkinson´s disease (IPD), Parkinson´s disease (PD), Lewy body disease (LBD) (the cohort contained 53 patients);
3. multiple system atrophy (MSA) group (the cohort contained 6 patients);
4. tauopathies group (progressive supranuclear palsy (PSP), corticobasal degeneration (CBD), frontotemporal dementia (FTD) (the cohort contained 16 patients);
5. other diagnoses group (including restless legs syndrome, polyneuropathies, hypokinetic-rigid syndrome, dopamine transporter deficiency) (the cohort contained 12 patients) and compared with 6) control group (HC) (vertebrogenic-algic syndrome, lumboischiadic syndrome) (the cohort contained 20 patients). The exclusion criteria for HC were the presence of neurodegenerative disease (in the patient or family history) or peripheral polyneuropathy.

We analyzed 58 male and 62 female patient samples. The average age of the patients was 67 years. The HC consisted of 13 males and 7 females samples. The average age of HC was 70 years (Table 1).

**Table 1:** Demographic and clinical characteristics of study cohorts

Group	*n* (M/F)	Age (mean, range)	Disease duration (years ± SD)	Diagnosis (dg.)
HC	20 (13/7)	70 (45–82)	-	no ND, no polyneuropathy
Park.sy.	33 (16/17)	70 (42–78)	3.64 ± 3.03	Prodromal / uncertain dg.
IPD + PD+ LBD	53 (29/24)	65 (34–83)	4.57 ± 3.87	Clinically confirmed α-synucleinopathy
MSA	6 (1/5)	67 (50–76)	4.17 ± 2.40	Clinically confirmed MSA
PSP + CBD + FTD	16 (9/7)	73 (56–79)	2.19 ± 1.68	Clinically confirmed tauopathy
Other diagnosis	12 (3/9)	59.5 (49–78)	3.27 ± 3.29	Neuropathies, restless legs syndrome, hypokinetic-rigid syndrome, dopamine transporter deficiency

Demographic data are shown as the number of subjects, sex distribution (male/female), mean age with range, and disease duration (mean ± SD, where available). Notes indicate relevant clinical features or diagnostic subcategories. The control group consisted of healthy individuals without a neurodegenerative disease or polyneuropathy

### Skin Biopsy

#### Sample Collection and Processing

**Fig. 2:**
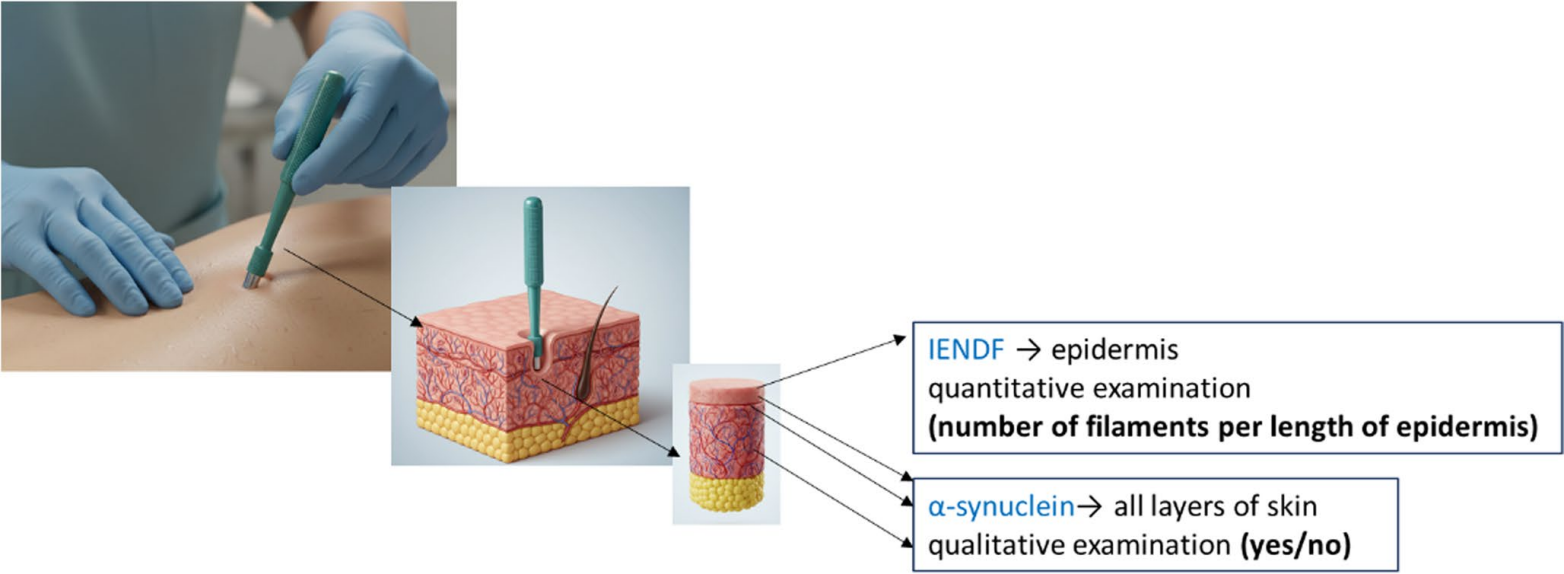
Skin punch biopsy procedure and evaluation workflow. Schematic representation of skin biopsies with a circular scalpel. In skin biopsies, after processing, we evaluated the quantitative amount of IENFD in the epidermis and the qualitative presence of p-α-syn in all skin layers. The image was created using AI-Gemini, an advanced AI-powered image creation tool

Prior to the outpatient excision biopsy using a circular scalpel (Fig. 2), local anesthesia was administered - typically 1% Mesocaine (alternative anesthetics were used in patients with Mesocaine allergy). The anesthetic was applied to the soft tissues (skin and subcutaneous tissue) at the biopsy sites on the back and the distal leg. No side effects of the biopsy procedure were reported.

The collected samples were then immersed in 4% paraformaldehyde and fixed for 4 h at room temperature. Then, the samples were placed in a buffer consisting of 10% sucrose in phosphate buffer for 24 h at a temperature of 2–4 °C. The next day, the samples were removed from the solution and immediately cut into Sect. 50 and 20 μm thick using a cryostat microtome (Leica CM1950, Leica Biosystems). The sections were cut directly onto glass microscope slides and were not processed as free-floating sections. The sections were then left to dry for 24 h on a plate at laboratory temperature. Then, they were transferred to an incubator (JOUAN, Innovens) where they were dried at a temperature of 47 °C. Finally, the slides were stored in histology boxes in a freezer at -20 °C until further immunohistochemical or immunofluorescence staining. All staining, incubation, washing, and mounting procedures were performed directly on the slides, using a coverslip and mounting medium for final preservation of the tissue sections.

#### Fluorescent Staining

Prior to incubation with fluorescent staining, the sections were slowly thawed at room temperature. Primary antibody incubation was performed as follows: the samples were fixed in 100% acetone: methanol at -20 °C for 10 min, rinsed with water for 5 min, and subsequently washed in phosphate-buffered saline containing 0.3% bovine serum albumine and 0.1% Tween 20 for 10 min. Afterwards, the slides were incubated with 3% normal goat serum for 30 min without further rinsing. Primary antibodies (raised in mouse) were then applied, mixed into a single tube, and diluted in the primary antibody diluent: PGP 9.5 (ThermoFisher Scientific), dilution 1:100 and purified anti-α-Synuclein phospho (Ser129) antibody (BioLegend), dilution 1:50. The sections were incubated with the primary antibody solution for 20 h at room temperature. Secondary antibody incubation followed rinsing in PBS (3 × 5 min) to remove residual primary antibodies. Secondary antibodies were mixed into one tube and diluted in the secondary antibody diluent: fluorescein isothiocynate-conjungate antibody (Millipore), dilution 1:100 and DyLight 550- conjungated antibody (ThermoFisher Scientific), dilution 1:500. The slides were incubated with the secondary antibody solution for 1 h at room temperature in the dark, rinsed in PBS (2 × 5 min), and finally rinsed with distilled water. The samples were then mounted in Mowiol mounting medium (Altium International).

The slides were evaluated using indirect immunohistochemistry at 200x magnification under a microscope (Olympus BX40, Olympus Corporation) with a fluorescent lamp (Olympus U-RFL-T, Olympus Corporation). The Olympus cellSens Standard program was used to evaluate and record the microscopic evaluation results.

The following features were evaluated: (1) the presence of p-α-syn in all layers of the skin – i.e. the epidermis, subepidermal tissue, and dermis. The evaluation was qualitative, with a yes/no (present/absent) result; (2) the density of thin fibers in the epidermis, with the result being the average number of thin fibers per length of epidermis.

According to the guidelines of the European Federation of Neurological Societies, PGP 9.5 positive nerve fibers at the epidermis-dermis junction were counted manually, and the length of the epidermal basement membrane was measured to calculate the intraepidermal nerve fiber density (IENFD; fibers/mm) (Lauria et al. 2010).

**Fig. 3:**
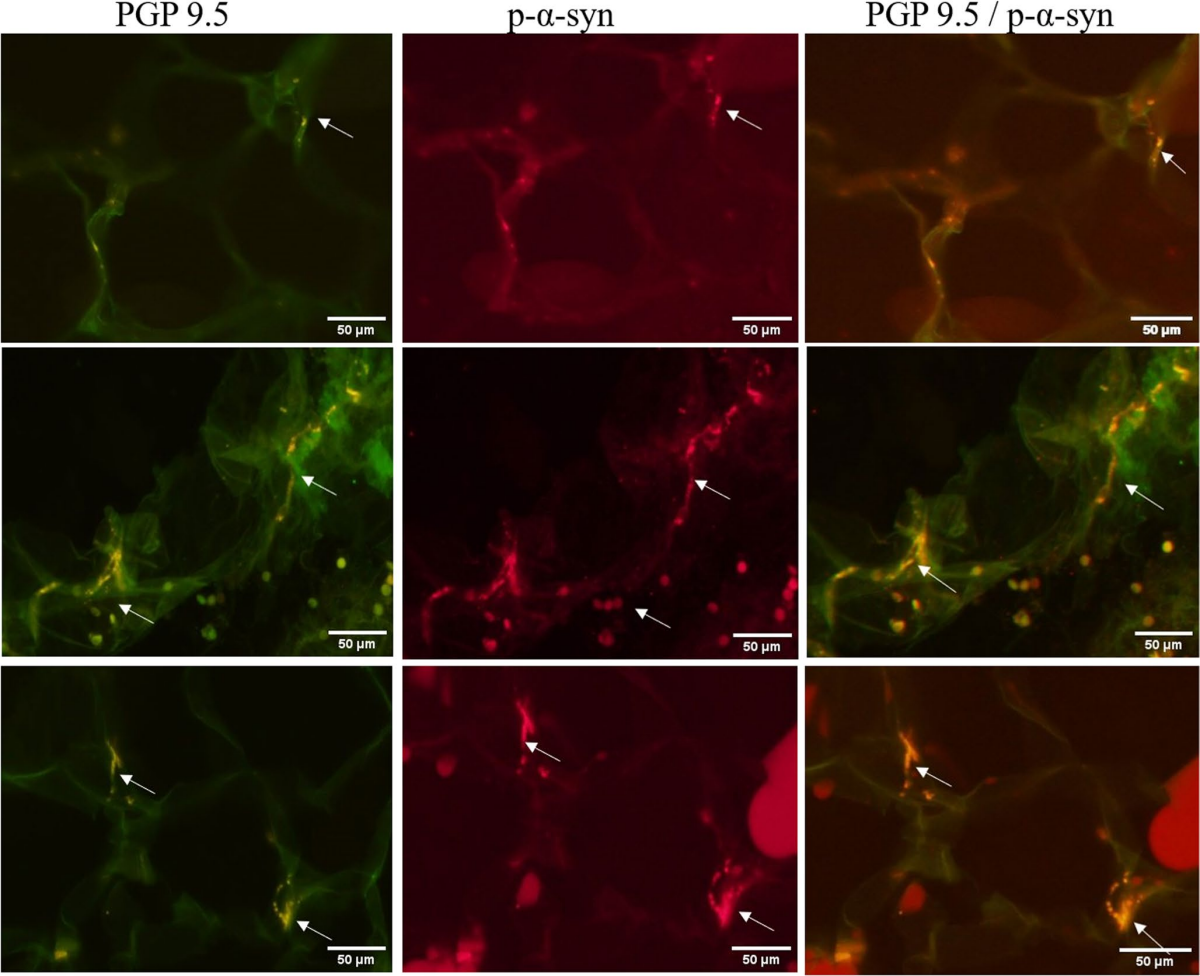
Cutaneous p-α-synuclein deposit detected by double immunofluorescence. Microscopic images of p-α-syn (marked with white arrows). The positive fibers present follow the course of the fiber and show very clear staining. The scale is 50 μm and the magnification is 200x. In most cases, the presence of p-α-syn is recorded only in the dermis, less frequently in the subepidermal space, and very rarely in the epidermis.s detected by double immunofluorescence

All samples were evaluated as standardized sections of 50 μm, as well as optimized sections of 20 μm thickness (Fig. 3). ImageJ software was used to evaluate and merge individual images.

#### Evaluation of the Presence of p-α-syn

The presence of p-α-syn was evaluated in all samples, both from the leg and the back.

#### Evaluation of IENFD Presence

The presence of IENFD was evaluated in leg samples with a slice thickness of 20 μm.

**Fig. 4:**
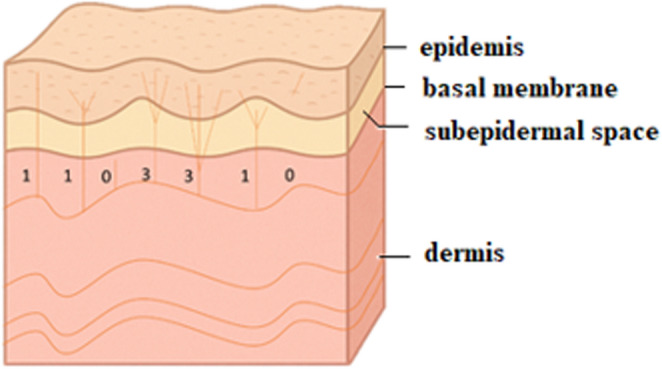
Principles of IENFD fiber counting

IENFD quantification was performed manually according to internationally accepted morphological criteria to ensure consistent identification of intraepidermal nerve fibers. In the microscopic field, the presence of IENFD was determined using the following rules:

1. Only nerves passing through the basement membrane into the epidermis are counted;
2. Nerves that branch before passing through the basement membrane are counted as two fibers;
3. Nerves that branch in the basement membrane are counted as two fibers;
4. Nerves that branch after crossing the basement membrane are counted as one fiber;
5. Fragments of nerves that pass through the basement membrane are counted;
6. Nerve fibers that approach the basement membrane but do not pass through it are not counted;
7. Epidermal nerve fragments that do not pass through the basement membrane are not counted (Fig. 4) (Bakkers et al. 2009).

These criteria ensured standardized fiber discrimination and minimized counting variability across samples.

IENFD was evaluated quantitatively in all skin biopsy samples according to established criteria. Individual nerve fiber counts were obtained for each patient and used for subsequent statistical analyses, including assessment of relationships with age and disease duration. For group-wise comparisons, IENFD results were additionally categorized as preserved or reduced, and the presence or absence of IENFD reduction was used for statistical evaluation across diagnostic groups.

#### Evaluation of Olfactory Test Results

**Graph 1:**
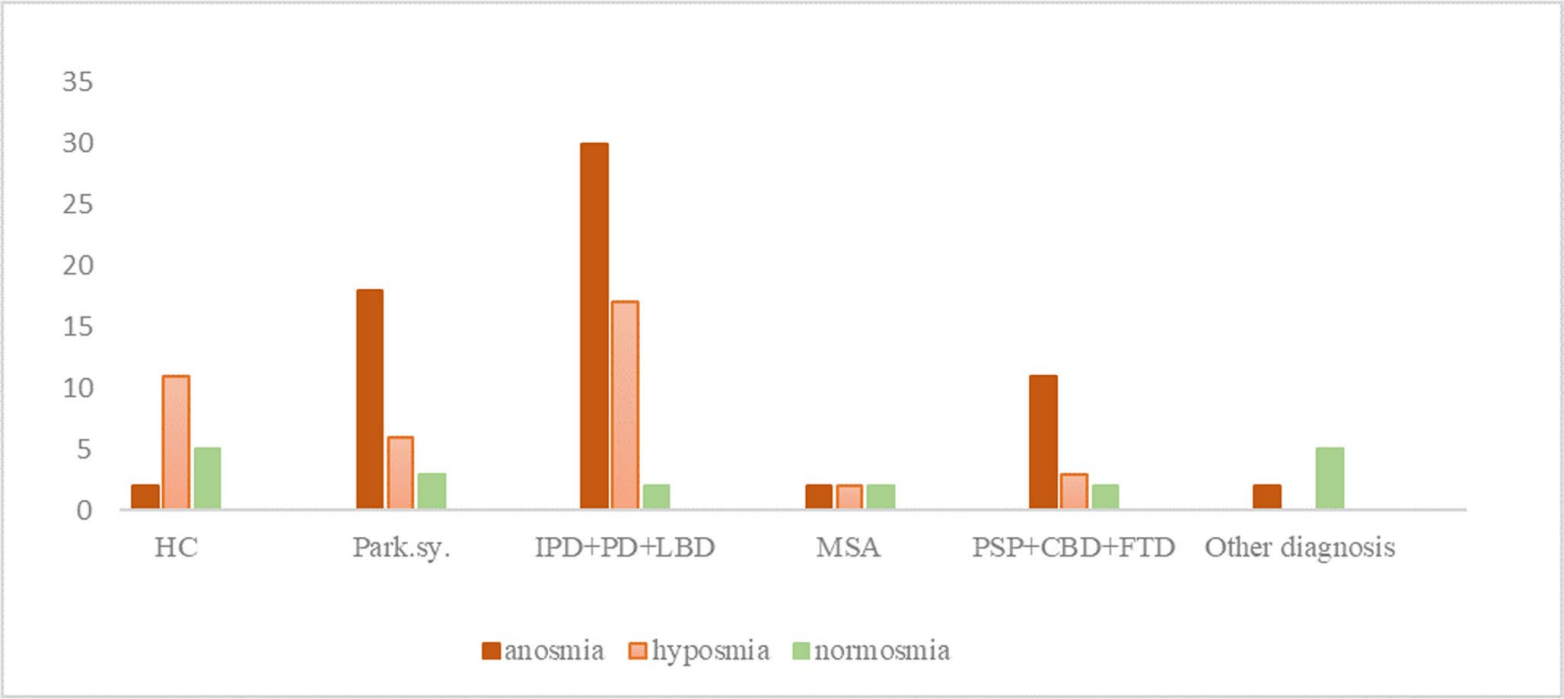
Olfactory performance in patient groups and controls. Graph illustrating the distribution of anosmia, hyposmia, and normosmia in healthy controls and patient groups. Anosmia was significantly more frequent in parkinsonian syndromes, IPD + PD + LBD, and tauopathies compared to controls

All patients and control group participants underwent a series of olfactory tests consisting of 12 synthetic odors (Screening 12 Test CZ LA-13-00001, MediSense). These tests were used to determine the preclinical development of neurodegenerative processes. The results of the olfactory tests were scaled as follows: 0 ≥ 6 correct answers correspond to anosmia, 6–10 to hyposmia, and 10 ≤ 12 to normosmia (Graph 1).

**Fig. 5:**
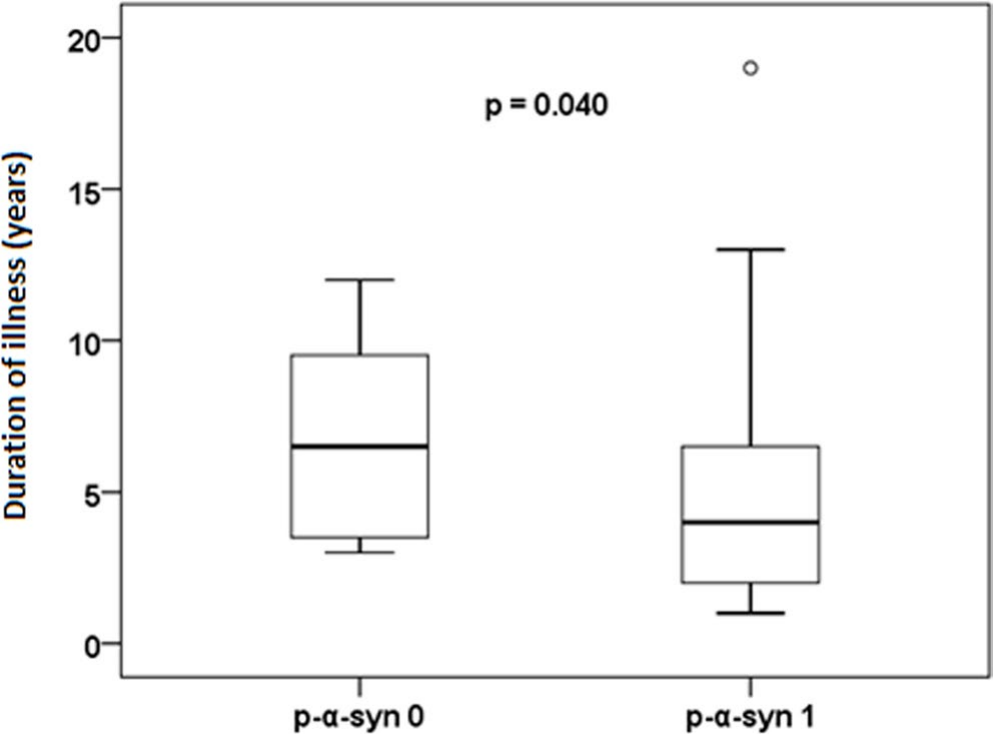
Relation between disease duration and p-α-syn positivity. Schematic representation of the relationship between the presence of p-α-syn and the duration of the disease. The average duration of the disease in the patient cohort was 4 years. p-α-syn 0 = patients without positive p-α-syn findings

## Results

### Statistical Analysis

Statistical examination of the results was performed using IBM SPSS Statistics software, version 23 (IBM Corp, Armonk, NY, USA). Quantitative parameters were compared using the Mann-Whitney U test. The Chi-square test or Fisher’s exact test was used to compare qualitative parameters. The normality of the data was assessed using the Shapiro-Wilk test. Multivariate logistic regression analysis with covariates age and sex was used to verify the significance of diagnosis for predicting the presence of alpha-synuclein deposition and the presence of IENFD. All tests were analyzed at a significance level of 0.05.

#### Evaluation of the Presence of p-α-syn

Compared to healthy controls (HC), a significantly higher incidence of p-α-syn positivity was demonstrated in all patient groups. Among the tauopathy and other diagnosis groups, a higher incidence of p-α-syn positivity was observed in the back (Table 2).

The results were also related to disease duration. Patients with a positive p-α-syn finding had a significantly shorter disease duration (*p* = 0.040) (Fig. 5).

#### Evaluation of IENFD Presence

A visible reduction or absence of IENFD was most prevalent in the tauopathy group (75%), while IENFD was most prevalent in the MSA group (83.3%) (Table 2).

The results were also compared with disease duration. No significant correlation was found between disease duration and IENFD presence (*p* = 0.910).

#### Evaluation of Olfactory Test Results

A significantly higher incidence of anosmia and a significantly lower incidence of hyposmia were demonstrated in the group of parkinsonian syndromes with tauopathy than in healthy controls (HC). Among α-synucleinopathies, the incidence of anosmia was significantly higher than in the HC group (Table 2)

**Table 2:** Statistical comparison of cutaneous and olfactory markers across diagnostic groups

(A)											
		HC		Park.sy.		IPD + PD+LBD		MSA		PSP + CBD+FTD	
		number	%	number	%	number	%	number	%	number	%
gender	male	13	65,0%	16	48,5%	29	54,7%	1	16,7%	9	56,3%
	female	7	35,0%	17	51,5%	24	45,3%	5	83,3%	7	43,8%
p-α-syn	yes	2	10,0%	20	60,6%	34	64,2%	6	100,0%	8	50,0%
	yes (leg)	1	5,0%	8	24,2%	11	20,8%	0	0,0%	3	18,8%
	yes (back)	0	0,0%	2	6,1%	5	9,4%	0	0,0%	3	18,8%
	no	17	85,0%	3	9,1%	3	5,7%	0	0,0%	2	12,5%
IENFD	yes	9	45,0%	19	57,6%	24	45,3%	5	83,3%	4	25,0%
	no	11	55,0%	14	42,4%	29	54,7%	1	16,7%	12	75,0%
olfactory test	anosmia	2	10,0%	18	54,5%	30	56,6%	2	33,3%	11	68,8%
	hyposmia	11	55,0%	6	18,2%	17	32,1%	2	33,3%	3	18,8%
	normosmia	5	25,0%	3	9,1%	2	3,8%	2	33,3%	2	12,5%
		Other diagnosis		Park.sy. vs. HC	IPD + PD+LBD vs. HC	MSA vs. HC	PSP + CBD+FTD vs. HC	Other dg. vs. HC			
		number	%								
gender	male	3	25,0%	1,000	1,000	0,326	1,000	0,142			
	female	9	75,0%								
p-α-syn	yes	4	33,3%	**< 0,0001**	**< 0,0001**	**0,001**	**0,0002**	**0,027**			
	yes (leg)	0	0,0%								
	yes (back)	3	25,0%								
	no	5	41,7%								
IENFD	yes	5	41,7%	1,000	1,000	0,848	1,000	1,000			
	no	7	58,3%								
olfactory test	anosmia	2	16,7%	**0,004**	**0,001**	1,000	**0,009**	0,065			
	hyposmia	0	0,0%								
	normosmia	5	41,7%								
(B)											
Dependent var.: presence of p-α-syn	p- value	adjOR	95%CI for adjOR								
group, ref =HC	<0.0001										
Park.sy.	**< 0.0001**	63.7	11.0 - 369.3								
IPD+PD+LBD	**< 0.0001**	103.5	18.3 - 585.6								
MSA	0.999	-	-								
PSP+CBD+FTD	**0.0002**	45.7	6.2 - 335.1								
other diagnosis	**0.010**	10.7	1.752 - 65.8								
sex male	0.413	1.635	0.504 - 5.207								
age	0.750	0.999	0.932 - 1.052								
(C)											
Dependent var.: presence of IENFD	p- value	adjOR	95%CI for adjOR								
group, ref =HC	0.318										
Park.sy.	0.404	1.640	0.513 - 5.249								
IPD+PD+LBD	0.959	1.029	0.351 - 3.015								
MSA	0.999	-	-								
PSP+CBD+FTD	0.120	0.306	0.069 - 1.360								
other diagnosis	0.778	0.804	0 177 - 3.657								
sex male	0.115	0.557	0 269 - 1.153								
Age	0.030	1.044	1 004 - 1.086								
Dependent var.: presence of IENFD	p- value	adjOR	95%CI for adjOR								
group, ref =HC	0.318										
Park.sy.	0.404	1.640	0.513 - 5.249								
IPD+PD+LBD	0.959	1.029	0.351 - 3.015								
MSA	0.999	-	-								
PSP+CBD+FTD	0.120	0.306	0.069 - 1.360								
other diagnosis	0.778	0.804	0 177 - 3.657								
sex male	0.115	0.557	0 269 - 1.153								
age	0.030	1.044	1 004 - 1.086								

Table summarizing the results described above. A) If a finding was positive in both cases – marked as yes; if p-α-syn was found only in the leg – marked as yes leg), and if only in the back – marked as yes (back). In the parkinsonian syndrome, α-synucleinopathy, and MSA groups, the proportion of yes positives was significantly higher. In the tauopathy group, the proportion of yes positives was significantly higher, as was the proportion of yes positives (back). Significant levels of IENDF were observed in the group of patients with MSA and Park.sy; B) In logistic regression, the presence of p-α-syn was analyzed as a dependent variable, with group (HC as reference), gender, and age as independent variables. The results confirm the data in Table 2 – patient groups (except MSA) are a significant predictor of the resence of p-α-syn and increase the likelihood of its detection compared to the control group. The comparison of MSA vs. HC was not statistically significant; C) In logistic regression, the presence of IENFD was analyzed as a dependent variable, with group (HC as reference), gender, and age as independent variables. The results confirm the data in Table 2—none of the patient groups represent a significant predictor of the presence of IENFD compared to the control group

## Discussion

Our data confirm that skin biopsy can serve as a minimally invasive tool for detecting p-α-syn and IENFD. The significantly higher incidence of p-α-syn in all patient groups compared to controls is consistent with prior reports demonstrating high sensitivity and specificity of cutaneous p-α-syn for α-synucleinopathies (Waqar et al. 2023; Donadio et al. 2021). However, interpretation of p-α-syn in skin sections must account for several methodological challenges. Thin sections may produce dot-like nerve profiles, making visual identification more difficult and increasing the risk of mistaking artifacts for true pathology. In most immunohistochemical staining methods, some degree of non-specific background noise is unavoidable. Wang et al. (2020) showed that certain dermal autonomic substructures, when not colocalized with protein gene product (PGP) fibers, may be misidentified as p-α-syn-positive. In line with these observations, our findings indicate that p-α-syn immunoreactivity in skin biopsies is not randomly distributed but shows a preferential localization within autonomic nerve fibers. The most frequent localizations were observed in perivascular nerve fibers surrounding small blood vessels and in nerve fibers innervating skin adnexal structures, whereas p-α-syn-positive deposits were less commonly identified in small dermal nerve bundles. This distribution pattern was consistently observed across patient groups and biopsy sites, supporting the concept that autonomic skin structures represent the most relevant targets for reliable detection of cutaneous p-α-syn. Notably, in our study, skin biopsies from the lower limb were consistently obtained from the distal part of the leg, and the higher incidence of p-α-syn positivity observed in paravertebral (proximal) sites suggests that proximal regions may provide increased detection rates, in line with previous reports indicating a site-dependent distribution of cutaneous p-α-syn (Donadio et al. 2021; Gibbons et al. 2023; Waqar et al. 2023). Consistently, p-α-syn deposits are typically found in autonomic skin structures such as sweat glands, pilomotor muscles, and blood vessels, but IHC alone may not reliably distinguish artifacts from genuine pathology, necessitating double-labeling of p-α-syn with a pan-axonal marker such as PGP 9.5 to ensure adequate specificity (Wang et al. 2020). On the other hand, Donadio et al. (2021) demonstrated that immunofluorescence staining yields excellent reproducibility in both synucleinopathy and non-synucleinopathy samples, reflecting the advantages of fluorescence techniques over conventional IHC. Yet even immunofluorescence is sensitive to pre-analytical variables: biopsy location, fixation techniques (Cassard et al. 2024), washing procedures, and removal of blood all influence staining quality. Hemoglobin can interfere with fluorescence signals and thus compromise interpretation (Moneriz et al. 2009). Similarly, certain preservation methods, including formalin fixation and paraffin embedding, may reduce both sensitivity and specificity. Importantly, our results showed p‑α-syn positivity not only in α-synucleinopathies but also in tauopathies and other neurodegenerative disorders, particularly within the PSP + CBS+FTD group. This observation can be explained by several factors. First, biological overlap between neurodegenerative proteinopathies may lead to the co-occurrence of α-syn and tau aggregates within the same neuronal populations or peripheral fibers (Han et al. 2022; Carrazana et al. 2025). Second, p‑α-syn may appear early in disease progression, preceding overt neurodegeneration, which could account for positivity in patients with tauopathies or preclinical changes (Zhao et al. 2025; Nolano et al. 2015). Third, methodological factors, such as the use of thin tissue sections, high antibody concentration, short incubation times, and absence of proteinase K digestion, may increase detection of non-aggregated α-syn or produce artifacts that appear positive (Delprete et al. 2025; Wang et al. 2020; Donadio et al. 2021). Together, these biological and technical considerations highlight the need for careful interpretation of p‑α-syn in non-α-synucleinopathy contexts. Compared to previous studies, the overall diagnostic accuracy of skin biopsy in our cohort was partly limited by reduced specificity, as reflected by p-α-syn positivity observed not only in synucleinopathies but also in other neurodegenerative disorders, particularly within the PSP + CBS+FTD group, and to a lesser extent in healthy controls. While this finding may partly reflect biological overlap between neurodegenerative proteinopathies or very early, subclinical pathological changes, technical aspects of the staining protocol likely also contributed. In our study, the relatively short primary antibody incubation period and the use of a comparatively high concentration of the anti-p-α-syn antibody may have favored non-specific binding and detection of native, non-aggregated α-synuclein, thereby reducing specificity. Recent evidence indicates that pretreatment with proteinase K (PK) can selectively remove soluble α-synuclein and significantly improve specificity by enhancing the detection of pathological, aggregated.

p-α-syn deposits (Delprete et al. 2025). The absence of PK digestion therefore represents a methodological limitation of the present study and highlights a potential strategy for improving diagnostic accuracy in future investigations. Notably, accumulating evidence suggests that the presence of α-syn aggregates in the skin parallels pathological progression in the brain (Carrazana et al. 2025; Han et al. 2022), supporting their relevance as peripheral biomarkers despite these technical limitations. In our cohort, preferential paravertebral positivity in tauopathies further highlights the importance of appropriate biopsy site selection. Importantly, p-α-syn positivity correlated with shorter disease duration, supporting the concept that cutaneous deposits may emerge early, potentially preceding overt neurodegeneration (Zhao et al. 2025; Nolano et al. 2015). This aligns with prior observations that p-α-syn aggregates may diminish in later disease stages due to clearance or neuronal loss. Consistent with previous studies, we did not observe any p-α-syn accumulation in the epidermis, suggesting that its detection may serve more as a temporal marker of active pathology rather than a static diagnostic hallmark. In contrast, our findings on IENFD diverge in part from the existing literature. Despite quantitative assessment of IENFD, absolute fiber counts did not yield statistically meaningful group differences. Therefore, results are presented as categorical presence or absence of IENFD reduction to allow clearer comparison between groups. While many studies report reduced IENFD in PD and related α-synucleinopathies (Jeziorska et al. 2019; Doppler et al. 2014; Kass-Iliyya et al. 2015), our cohort showed the most pronounced reduction in tauopathies (75%), whereas IENFD remained largely preserved in MSA (83.3%). This preservation contrasts with earlier reports of cutaneous denervation in MSA and PSP (Dubbosio et al. 2021). Potential explanations include disease-specific peripheral nerve involvement, methodological differences (e.g., section thickness, fixation), or the limited sample size, particularly in the MSA group (*n* = 6), which reduces statistical power and generalizability. Interestingly, we found no significant correlation between IENFD and disease duration, in contrast to findings of progressive decline over time (Doppler et al. 2014; Yuan et al. 2024). This discrepancy may reflect heterogeneous progression patterns, compensatory mechanisms, or sampling-related variability. Longitudinal studies are therefore needed to determine whether cutaneous fiber loss differs among neurodegenerative subtypes. Adding olfactory testing improved diagnostic discrimination, supporting prior evidence that anosmia is a frequent non-motor symptom in both α-synucleinopathies and tauopathies (Ehler 2012; Gummerson et al. 2025). The occurrence of hyposmia among controls suggests possible preclinical neurodegeneration, consistent with observations from prospective cohorts (Han et al. 2022). Overall, our findings highlight both the promise and the challenges of cutaneous biomarkers. While p-α-syn shows potential as an early disease marker and olfactory testing enhances diagnostic differentiation, IENFD patterns remain heterogeneous. Technical variability, particularly in staining and sample preparation, remains a major contributor to inconsistent findings. Therefore, methodological standardization and large multicenter validation studies are essential for establishing the clinical utility of these approaches in routine diagnostics (Carrazana et al. 2025; Cassard et al. 2024).

## Conclusion

Our study provides compelling evidence that skin biopsy is an effective and minimally invasive tool for diagnosing neurodegenerative diseases. The detection of p-α-syn serves as an early marker of pathological processes, and the quantification of IENFD reflects small fiber degeneration, which is most pronounced in multiple system atrophy and parkinsonian syndromes. Combined with olfactory testing, these cutaneous markers significantly improve the discrimination of α-synucleinopathies, tauopathies, and controls. Together, these markers support the integration of peripheral tissue biomarkers into multimodal diagnostic algorithms and next-generation criteria for neurodegenerative diseases (e.g., SynNeurGe, NSD-ISS). Future research should focus on methodological standardization and validation in larger, multicenter cohorts to establish the full clinical utility of these biomarkers.

## Data Availability

Detailed information is provided in Table 1-2, Graph 1 and Figs. 1, 2, 3, 4 and 5 used in this study. All tables and figures are enclosed.
