# Correction: Cutaneous α-Synuclein Pathology as a Differential Marker: A Histological and Statistical Comparison across Neurodegenerative Disease Groups

**DOI:** 10.1007/s12031-026-02515-y

**Published:** 2026-04-27

**Authors:** Dorota Šebelová, Kateřina Menšíková, Michaela Kaiserová, Lenka Satke, Zuzana Grambalová, Kateřina Čížková, Zdeněk Tauber, Kateřina Klíčová, Dominik Hraboš, Sarah E. V. Cook, Jana Zapletalová, Petr Kaňovský

**Affiliations:** 1 Department of Neurology, Faculty of Medicine and Dentistry, Palacky University, Olomouc, 77900 Czech Republic; 2 Department of Neurology, Olomouc University Hospital, Olomouc, 77900 Czech Republic; 3 Department of Histology and Embryology, Faculty of Medicine and Dentistry, Palacky University, Olomouc, 77900 Czech Republic; 4 Department of Molecular Pathology, Faculty of Medicine and Dentistry, Palacky University, Olomouc, 77900 Czech Republic; 5 Olomouc Brain Bank, Department of Neurology and Department of Clinical and Molecular Pathology, University Hospital Olomouc and Faculty of Medicine and Dentistry, Palacky University, Olomouc, Czech Republic; 6 Department of Medical Biophysics, Olomouc University Hospital, Olomouc, Czech Republic

**Correction: Journal of Molecular Neuroscienc** **e (2026) 76:37**

**https://doi.org/10.1007/s12031-026-02486-0**

The authors of the paper “Cutaneous α-Synuclein Pathology as a Differential Marker: A Histological and Statistical Comparison across Neurodegenerative Disease Groups” have requested for a modification. Specific revisions are detailed as follows:

The co-author’s name is incorrectly stated in the header—instead of Sarah E.V. Cook, it says Sara E.V. Cook—but it is correct in Contribution, and it was correct and checked in the proofs.

Also, dedications to grants in the article has been added.

The original article has been corrected.

